# Bullous Pemphigoid in Children: Sustained 8‐Year Remission After Short‐Course Corticosteroid Therapy

**DOI:** 10.1002/ccr3.71882

**Published:** 2026-02-11

**Authors:** Fengchang Wang, Hongxia Li, Yue Xu, Wenge Wang

**Affiliations:** ^1^ Department of Pediatrics Air Force Characteristic Medical Center Beijing China

**Keywords:** bullous pemphigoid, case report, child, long‐term follow‐up

## Abstract

This article reports a case of bullous pemphigoid (BP) in a 5‐year‐old child who achieved sustained remission for up to 8 years following short‐course systemic corticosteroid therapy, aiming to provide observational evidence for the clinical management of childhood BP. The child initially presented with pruritic rashes and blisters, which were once misdiagnosed as infectious rashes; finally, the diagnosis was confirmed by direct immunofluorescence (DIF), histopathology, and other examinations. After treatment with systemic glucocorticoids combined with anti‐infective therapy, the skin lesions basically resolved within 2 weeks, and the tapering and discontinuation of drugs were completed within 26 weeks of treatment. During the 8‐year follow‐up period, no recurrence or treatment‐related complications were observed. This case suggests that although childhood BP is prone to misdiagnosis, it responds well to glucocorticoid treatment, and long‐term remission can be achieved with a relatively short treatment course; however, this model still needs to be verified by large‐sample studies.

## Key Clinical Message

This article reports a case of childhood bullous pemphigoid (BP) with an 8‐year long‐term follow‐up. Although the patient was initially misdiagnosed, she exhibited a favorable response to systemic corticosteroid treatment. After receiving treatment, the child achieved sustained remission and remained recurrence‐free for 8 years.

## 1 Introduction

Bullous pemphigoid (BP) is a typical autoimmune subepidermal bullous disease, with the pathological core being an autoimmune response targeting the basement membrane zone (BMZ) antigens BP180 and BP230 [1]. It predominantly affects the elderly, with a global cumulative incidence of approximately 8.2 cases per million population [2]. The disease is rare in the pediatric population, and relevant epidemiological data are limited. A 2015 German study on the prevalence of autoimmune blistering diseases in children reported that the prevalence of BP in individuals under 18 years old was 4.9 cases per million population [3]. Current knowledge about BP is mainly based on elderly patients; advanced age, neurological diseases, and specific medications (e.g., DPP‐4 inhibitors) are recognized as major risk factors [4]. However, the role of these factors is limited in children, and whether the clinical characteristics, treatment response, and long‐term prognosis of childhood BP have unique features remains to be further explored. Existing literature on childhood BP mostly consists of case reports and lacks prospective long‐term follow‐up data. A search in the PubMed database identified only one single‐center case series reporting follow‐up experience exceeding 10 years [5]. Due to the scarcity of long‐term, high‐quality cohort data, there are currently no evidence‐based clinical diagnosis and treatment guidelines specifically for childhood BP [6]. This insufficiency in the evidence base may hinder the optimization and standardization of clinical management strategies for childhood BP.

This article reports a case of BP in a previously healthy 5‐year‐old female child. The child was misdiagnosed multiple times at the initial visit; however, after standardized glucocorticoid treatment, the skin lesions resolved rapidly, and physiological remission without recurrence was maintained for 8 years after drug discontinuation. Through long‐term follow‐up of this case, this study aims to provide observational evidence for the clinical characteristics and management of childhood BP and emphasizes the necessity of in‐depth research on childhood BP as a unique subtype.

## 2 Case History/Examination

**FIGURE 1:**
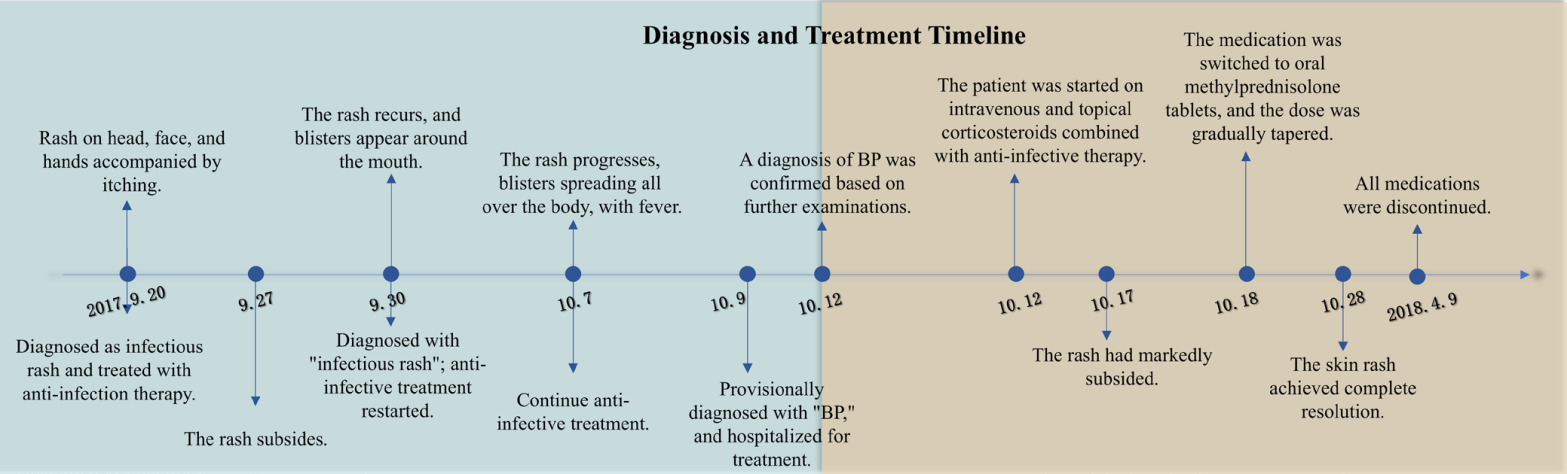
Diagnosis and treatment course of the child. This figure illustrates the complete clinical course from the onset of initial symptoms to complete remission.

**FIGURE 2:**
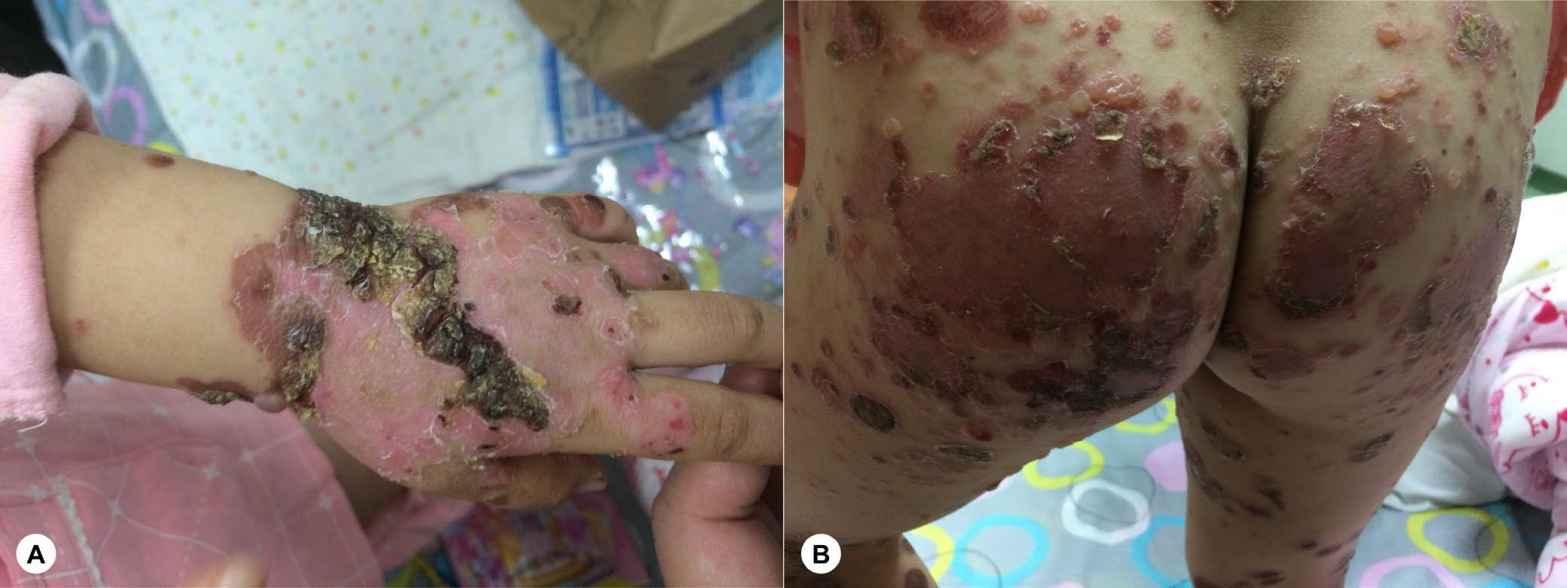
Clinical manifestations of the child's skin lesions. (A) Multiple tense bullae, erosion, and crusting are visible on the dorsal hands. (B) Extensive erythema, multiple tense bullae, erosive surfaces, and honey‐yellow crusting are distributed over the buttocks and thighs.

A 5‐year‐5‐month‐old female child had no previous medical history. She was admitted to the hospital due to “generalized rash with blisters for more than half a month and fever for 3 days.” There was no special family medical history, and she had not used any medications. Half a month before admission, the child developed red rashes on the head, face, and hands without a clear cause, accompanied by significant pruritus, but no fever or blisters. She was seen at another hospital, where a tentative diagnosis of “infectious rash” was made, and she received anti‐infective treatment such as oral cefalosporins (treatment details from an external hospital unavailable). One week after medication administration, the rash gradually subsided. Three days after discontinuing the medication, the child's condition relapsed: a rash reappeared on the back, which quickly spread to the perioral area and buttocks, with blisters around the mouth and significant pruritus; her body temperature remained normal during this period. During the follow‐up visit, the local hospital still considered the possibility of “infectious rash,” continued anti‐infective treatment (treatment details from an external hospital unavailable), and added oral anti‐allergic drugs and topical glucocorticoids. One week after treatment, the child's rash significantly worsened compared with before: blisters spread from the perioral area to the entire body, accompanied by severe pruritus. Three days before admission, the child developed a high fever, with the maximum body temperature reaching 39.5°C. Despite continuing the above‐mentioned treatment and adding antipyretic drugs, the child's body temperature still fluctuated, and the rash and blisters progressively worsened, gradually spreading to the entire body. For further treatment, she was seen at the pediatric outpatient department of our hospital and admitted with a tentative diagnosis of “bullous disease?” (see Figure 1). The child's general condition was good. Specialist physical examination revealed scattered diffuse erythema and maculopapules all over the body, with multiple tense blisters on them; the blister fluid was clear, and some blisters had ruptured and formed crusts (Figure 2). Nikolsky sign (both direct and indirect methods) was negative. No mucosal involvement was observed in the perineal and perianal regions. The Bullous Pemphigoid Disease Area Index (BPDAI) score was 69.9.

## 3 Methods

**FIGURE 3:**
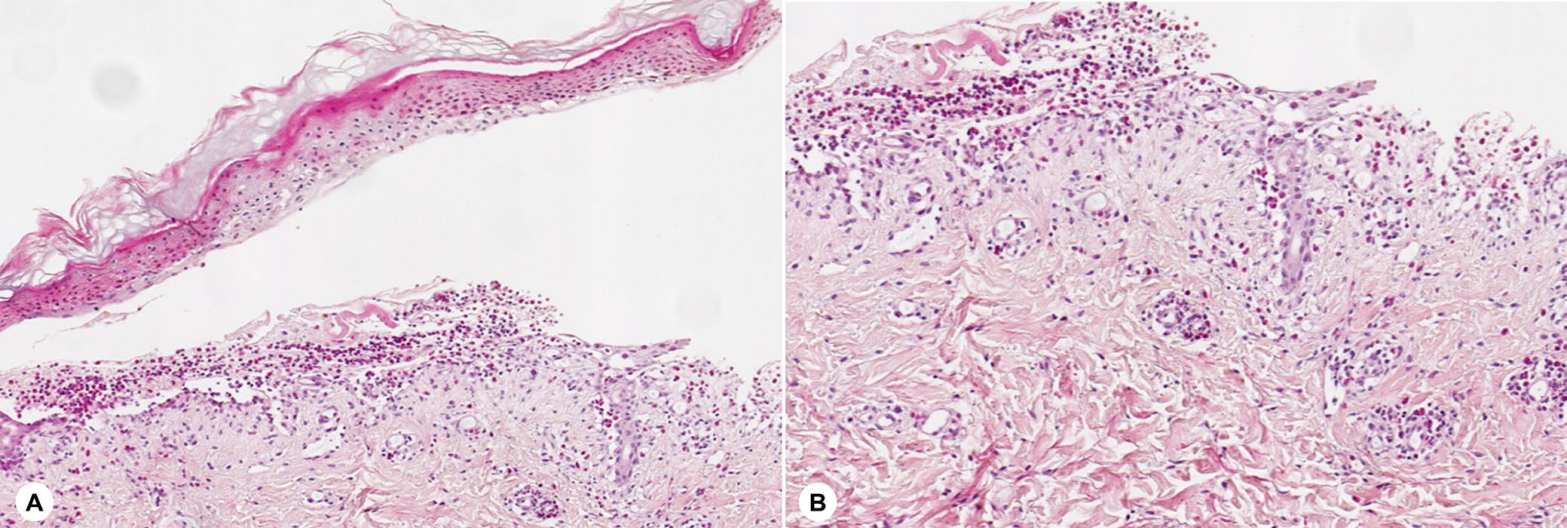
Histopathological findings of the child's skin lesions. (A) Subepidermal blister formation is observed. (B) A large number of eosinophils and lymphocytes' infiltration is seen under high‐power field.

After admission, the child underwent a comprehensive set of examinations, with results as follows: complete blood count (CBC) showed a white blood cell (WBC) count of 15.41 × 10^9^/L and a C‐reactive protein (CRP) level of 5 mg/L; eosinophil count: 1.54 × 10^9^/L; immunoglobulins: IgG 8.96 g/L, IgA 1.19 g/L, IgM 0.85 g/L; erythrocyte sedimentation rate (ESR): within the normal range; Blood culture showed no bacterial growth; Blister fluid culture revealed *Staphylococcus aureus* (a small amount of Gram‐positive cocci were observed on smear); dermoscopy showed incomplete epidermis, accompanied by erosion, crusting, and blisters; histopathological examination revealed epidermal edema, intraepidermal and subepidermal blisters, a large number of eosinophils in the blister cavity, and inflammatory infiltration dominated by lymphocytes and eosinophils around the superficial dermal blood vessels (Figure 3); direct immunofluorescence (DIF) revealed linear deposition of IgG and C3 along the basement membrane zone (BMZ) (specimen obtained from normal skin within 1 cm of the blister margin). Serological testing indicated elevated titers of BP180 and BP230 antibodies: BP180 antibody was 100.00 U/mL (reference range: 0–9 U/mL), and BP230 antibody was 12.01 U/mL (reference range: 0–9 U/mL). Based on the child's clinical manifestations and auxiliary examination results, a diagnosis of “BP” was confirmed. Subsequently, the child was administered intravenous methylprednisolone sodium succinate (1 mg/kg/day) and topical mometasone furoate cream plus tacrolimus ointment. Due to the significantly elevated WBC count, concurrent infection was suspected, so piperacillin‐tazobactam (100 mg/kg every 8 h) was added for anti‐infective treatment. The child's clinical symptoms improved markedly, thus intravenous medications were discontinued and switched to oral methylprednisolone tablets. The glucocorticoid tapering regimen was formulated with reference to China's *Expert Recommendations for the Diagnosis and Treatment of Bullous Pemphigoid (2016)* and adjusted according to the changes in the child's clinical symptoms. Specifically, the initial dose was 1 mg/kg/day, with a reduction of 0.1 mg/kg/day every week; after 6 weeks, the reduction was adjusted to 0.05 mg/kg/day every 2 weeks. When the dose was tapered to 0.1 mg/kg/day, this dosage was maintained for 4 weeks before drug discontinuation, with a total treatment course of 26 weeks.

## 4 Conclusions and Results

After treatment with systemic glucocorticoids, the child's skin lesions significantly alleviated within 5 days and eventually resolved gradually within 2 weeks. All medications were gradually discontinued within 26 weeks in accordance with the treatment plan. During the follow‐up visits at 1 month, 6 months, 12 months, 2 years, 4 years, 6 years, and 8 years after the initiation of treatment, no treatment‐related complications or recurrence symptoms were observed. The child's growth and development were consistent with those of healthy peers of the same age, and no obvious glucocorticoid‐related adverse reactions (such as growth retardation, abnormal weight gain) were detected.

## 5 Discussion

This article reports a rare case of childhood BP with long‐term follow‐up. BP is an autoimmune subepidermal bullous disease that primarily affects the elderly, and its occurrence in children is extremely rare. The core mechanism of this disease is an immune attack driven by autoantibodies against BP180 and BP230. Through the dual hits of complement activation to chemotact inflammatory cells and direct induction of keratinocyte activation, these antibodies synergistically disrupt the basement membrane zone (BMZ), ultimately leading to the formation of characteristic subepidermal blisters [7]. However, the initiating factors that trigger autoantibody production and the specific mechanisms underlying immune dysregulation have not yet been fully elucidated.

Studies have shown that BP has diverse predisposing factors, including genetic, environmental, autoimmune, and drug‐related factors. Currently, there are no large‐scale studies on the predisposing factors of childhood BP; only a small number of case reports suggest that its onset may be associated with vaccination [8]. However, due to the limited sample size, these views still require further evidence to support them. In the case of this child, there was no history of vaccination or special medication use within several months before the onset of the disease, so vaccine‐related and drug‐related predisposing factors can be temporarily ruled out.

BP typically presents with eczematous or urticarial rashes initially, followed by tense bullae accompanied by severe pruritus. The rashes are most commonly distributed on the flexor surfaces of the extremities, abdomen, waist, and forearms. Approximately 10%–20% of patients may have mucosal involvement, with the oral cavity being the most frequent site [9]. Previous literature reports indicate that the clinical manifestations of childhood BP are generally similar to those of adult BP; however, some cases suggest that pediatric patients may have skin involvement on the hands and feet [8, 10]. The child in this case also exhibited skin lesions on the hands and feet, implying potential differences in clinical features between childhood BP and adult BP. Some patients may not present with typical bullae for several months to years before a definitive BP diagnosis is made [1]. In the early stage of this case, the child also had no blisters or bullae; during two medical visits, the child was misdiagnosed as having an “infectious rash” and received anti‐infective treatment. Such misdiagnoses are not uncommon, primarily because the prodromal skin lesions of BP lack specificity and their clinical manifestations are often similar to those of drug eruptions, papular urticaria, or nonspecific infectious skin diseases, which easily lead to delayed diagnosis. Therefore, for children with atypical rashes, the possibility of BP should be considered, and timely differentiation from diseases such as pemphigus vulgaris and linear IgA bullous dermatosis should be performed via histopathological examination and DIF (see Table 1).

**TABLE 1:** Differential diagnosis of childhood bullous pemphigoid.

Disease	Key differentiating points
*Non‐bullous diseases*	
Urticaria [11]	Recurrent wheal‐like rashes and angioedema, accompanied by pruritus. Pathological examination reveals dermal edema with lymphocyte and eosinophil infiltration, but no vasculitis. Antihistamine therapy is effective
Atopic dermatitis/Eczema [12]	Presents as chronic, recurrent, pruritic eczematous lesions, with lichenification, scaling, dryness, and fissures observable. Th2 immune skew and elevated IgE levels are present
Drug eruption [13]	Appears rapidly within minutes to hours after drug administration, with diverse morphologies. It is usually an IgE‐mediated type I hypersensitivity reaction, but non‐immunological pathways may also be involved. The oral drug provocation test is the gold standard for confirming this disease
Scabies [14]	Caused by Sarcoptes scabiei. Typical symptoms include red papules, burrows, and nodules in thin and tender skin areas. Diagnosis can be confirmed by finding mites or mite eggs in skin scrapings
*Bullous diseases*	
Linear IgA Bullous Dermatosis (LAD) [15]	Pediatric LAD tends to occur in perioral, inguinal, and other regions, characterized by annular tense blisters with a “beaded” edge. DIF reveals linear deposition of IgA along the basement membrane zone
Acquired Epidermolysis Bullosa (EBA) [16]	The classic type presents with fragile blisters and scars in friction‐prone areas, while the inflammatory type shows widespread blisters. Diagnosis relies on DIF demonstrating linear deposition of IgG/C3 along the basement membrane zone, and salt‐split skin test showing antibodies localized to the dermal side
Bullous Impetigo [17]	Caused by local infection with *Staphylococcus aureus* , presenting as flaccid bullae with indistinct surrounding erythema. The blister fluid changes from clear to turbid, and characteristic honey‐yellow crusts remain after blister rupture; *Staphylococcus aureus* can often be detected in bacterial culture of the lesion
Staphylococcal Scalded Skin Syndrome (SSSS) [17]	A systemic toxemia caused by *Staphylococcus aureus* . Its clinical features include diffuse erythema, extensive epidermal detachment, positive Nikolsky sign, and systemic toxic symptoms. Bacterial culture of the skin lesions is usually negative
Bullous Systemic Lupus Erythematosus (BSLE) [18]	Presents with widely distributed tense bullae, which tend to occur on the head and neck, extremities, trunk, and mucous membranes. DIF shows linear deposition of IgG/C3 along the basement membrane zone, and pathology reveals subepidermal bullae mainly composed of neutrophils

According to the updated clinical practice guidelines of the European Academy of Dermatology and Venereology (EADV) in 2022, the diagnosis of BP should be based on typical clinical manifestations, combined with the results of DIF testing of the skin BMZ and the detection of serum‐specific autoantibodies (e.g., BP180 and BP230 antibodies) for comprehensive judgment. DIF is the gold standard for diagnosis, with the typical manifestation of linear deposition of IgG and/or C3 along the BMZ; the child in this case conformed to this feature. Serological antibody detection is often used for screening of highly suspected BP cases and monitoring of disease activity [9]. Although the EADV guidelines do not list histopathology as a mandatory diagnostic item, it still holds important value in clinical practice, primarily for ruling out other subepidermal bullous diseases.

Currently, the treatment of BP mainly relies on glucocorticoids. For patients with mild‐to‐moderate BP, potent topical glucocorticoids are the first choice. After reaching the Controlled Disease Activity (CDA; defined as no new skin lesions, cessation of pruritus, and healing of old skin lesions), the treatment is maintained for 15 days, followed by gradual tapering and discontinuation within 4 months. For patients with extensive skin lesions, oral prednisone (0.5–0.75 mg/kg/day) can be administered; 15 days after achieving CDA, the dose is gradually reduced, with a usual treatment course of 3–12 months. Doxycycline, dapsone, or methotrexate can be used as alternative or adjuvant therapies. Throughout the entire treatment course, dynamic adjustments should be made based on the BPDAI score and anti‐BP180 antibody titer to prevent recurrence. For patients with contraindications to or dependence on glucocorticoids, doxycycline, dapsone, or immunosuppressants are preferred [9]. However, there are currently no specific treatment guidelines for childhood BP; the existing treatment of childhood BP mostly refers to adult regimens. Nevertheless, the data on their efficacy and safety are mainly derived from small‐sample case series or individual case reports, lacking support from long‐term follow‐up. In this case, after the child reached CDA, glucocorticoid treatment was completed within 26 weeks, which was shorter than the standard adult regimen. During the subsequent 8‐year follow‐up, the child's condition remained stable without recurrence. This is similar to the case reported by Ana María Sáenz et al. [5] (a 15‐month‐old infant recovered after 27 days of treatment and had no recurrence during a 10‐year follow‐up). A small‐sample study indicated that the average disease duration of pediatric patients is approximately 14 months (range: 1.5 months to 5 years) [19]. Existing evidence suggests that childhood BP may have a shorter disease duration and better prognosis compared with adult BP [6], which may support shorter‐course individualized treatment strategies to reduce glucocorticoid‐related side effects. However, since most of the data are derived from individual cases, further verification through large‐sample studies is still required.

The prognosis of BP is a key focus in clinical practice. A 15‐year retrospective analysis of 1018 Chinese patients conducted by Shan Cao et al. revealed that BP is associated with relatively high recurrence and mortality rates: the cumulative recurrence rates at 1 year, 3 years, and 5 years were 21.9%, 46.6%, and 60.9%, respectively; the all‐cause mortality rates were 22.8%, 31.2%, and 34.5%, respectively. Additionally, 573 patients (56.3%) experienced treatment‐related adverse events requiring intervention [20]. These findings indicate that adult BP often has a protracted course and is prone to recurrence. However, data on the prognosis of childhood BP remain extremely limited. The favorable outcome of this child—who maintained sustained remission over an 8‐year follow‐up period—provides valuable support for the notion that childhood BP may have a more benign prognosis. However, this study has obvious limitations: no regular laboratory index monitoring was performed during the follow‐up period, and the conclusions are limited by the nature of a single‐case report. Therefore, caution should be exercised when extrapolating these conclusions.

In the future, large‐scale, long‐term prospective studies are urgently needed to systematically clarify the epidemiological characteristics of childhood BP and establish dedicated treatment regimens and prognosis assessment systems for this population.

## Author Contributions

**Fengchang Wang:** conceptualization, data curation, formal analysis, investigation, methodology, validation, visualization, writing – original draft, writing – review and editing. **Hongxia Li:** conceptualization, data curation, formal analysis, investigation, project administration, validation, visualization, writing – original draft, writing – review and editing. **Yue Xu:** investigation, methodology, validation, visualization, writing – review and editing. **Wenge Wang:** conceptualization, methodology, project administration, resources, supervision, validation, writing – review and editing.

## Funding

The authors have nothing to report.

## Ethics Statement

This article is a case report, so approval from the institutional ethics committee of our unit is not required. Meanwhile, we have obtained written informed consent from the patient's legal guardian.

## Conflicts of Interest

The authors declare no conflicts of interest.

## Data Availability

Data sharing not applicable to this article as no datasets were generated or analyzed during the current study.
